# A retrospective molecular study of *Cryptosporidium* species and genotypes in HIV-infected patients from Thailand

**DOI:** 10.1186/s13071-019-3348-4

**Published:** 2019-03-12

**Authors:** Anna Rosa Sannella, Yupin Suputtamongkol, Ekkarat Wongsawat, Simone M. Cacciò

**Affiliations:** 1 Department of Infectious Disease, Istituto Superiore di Sanità, Rome, Italy; 2 Department of Medicine, Faculty of Medicine, Siriraj Hospital, Mahidol University, Bangkok, Thailand

**Keywords:** *Cryptosporidium*, HIV, Thailand, Molecular typing, Species, Subtypes

## Abstract

**Background:**

Opportunistic infections represent a serious health problem for HIV-infected people. Among enteric infections, cryptosporidiosis, a severe and life-threatening diarrheal disease, is of particular importance in low economic settings where access to anti-retroviral therapy is limited. Understanding transmission routes is crucial in establishing preventive measures, and requires the use of informative genotyping methods. In this study, we performed a retrospective analysis of *Cryptosporidium* species in 166 stool samples collected from 155 HIV-infected patients during 1999–2004 at the Siriraj Hospital in Bangkok, Thailand.

**Results:**

Microscopic examination of stools identified 104 of the 155 patients as positive for *Cryptosporidium*. Other common pathogens identified were microsporidia, *Isospora*, *Giardia*, *Strongyloides* and *Opisthorchis.* All samples were tested by amplification of a fragment of the *18S* rDNA locus, and sequencing showed the presence of *Cryptosporidium hominis* (*n* = 42), *C. meleagridis* (*n* = 20), *C. canis* (*n* = 12), *C. felis* (*n* = 7), *C. suis* (*n* = 6) and *C. parvum* (*n* = 5). Genotyping at the glycoprotein 60 (*gp60*) locus revealed substantial variability in isolates of *C. hominis* and *C. meleagridis*. Among *C. hominis* isolates, subtype IeA11G3T3 was the most prevalent, but allelic family Id was the more diverse with four subtypes described, two of which were identified for the first time. Among *C. meleagridis* isolates, seven subtypes, two of which were new, were found in the allelic family IIIb, along with new subtypes in allelic families IIIe and IIIg. In the four *C. parvum* isolates, subtype IIoA16G1, a rare subtype previously reported in a Swedish patient who had traveled to Thailand, was identified.

**Conclusions:**

This study confirms the high susceptibility of HIV-infected individuals to infection with different *Cryptosporidium* species and subtypes, and further stresses the importance of surveillance for opportunistic intestinal protozoans.

**Electronic supplementary material:**

The online version of this article (10.1186/s13071-019-3348-4) contains supplementary material, which is available to authorized users.

## Background

The HIV/AIDS epidemic is still a major public health problem, and the Southeast Asia region, where about a quarter of the world’s population live, is second only to sub-Saharan Africa in terms of disease burden [1]. In the 11 member states of the Southeast Asia region, there are an estimated 3.5 million people living with HIV [1]. Thailand is the only country with an HIV prevalence of over 1% among the adult population (aged 15–49 years), although a higher prevalence is reported among sex workers and their clients, men who have sex with men, people who inject drugs and transgender individuals.

Opportunistic infections still represent a serious threat for HIV-infected individuals, and among these, *Cryptosporidium* is recognized as a leading cause of prolonged, severe diarrheal disease, accounting for up to a third of diarrhea cases in HIV patients [2, 3]. Due to the lack of effective drugs to treat cryptosporidiosis, avoiding exposure to the parasite and maintaining immune competence are the only ways to prevent infection in these patients [4].

A recent global systematic review and meta-analysis of *Cryptosporidium* infection in HIV-infected people identified 106 studies with 43,218 patients from 36 countries examined [5]. Among the studies, nine were from Asia and the Pacific and eight from Thailand, where a pooled prevalence of 15.2% (95% CI: 8.8–21.6%) was inferred from 947 HIV-infected people [5].

Human cryptosporidiosis is mainly due to *Cryptosporidium hominis* and *C. parvum*, but many other species of potential zoonotic origin are known to infect both immunocompetent and immunocompromised people [3]. Therefore, information about the circulating parasite species and genotypes is essential in understanding the transmission dynamics of cryptosporidiosis. Moreover, studies have suggested that the clinical manifestations of cryptosporidiosis are influenced by the species or the genotypes involved [6, 7], therefore strengthening the value of informative genotyping data.

Thus far, molecular characterization of parasite isolates from HIV-infected people in Thailand has resulted in the identification of *Cryptosporidium hominis*, *C. parvum*, *C. meleagridis*, *C. felis*, *C. canis* and *C. muris* [8–12].

In this study, we investigated *Cryptosporidium* species and genotypes in 166 archived stool samples collected during 1999–2004 from 155 HIV-infected individuals at the Siriraj Hospital in Bangkok.

## Methods

### Study design

The investigation originally aimed at determining the etiologic cause(s) of diarrhea in HIV-infected patients [13]. Diarrhea was defined as the passage of at least two watery or loose stools per day, while chronic diarrhea as the continuous presence of diarrhea for more than 3 weeks. Patients attending the outpatient clinic at Siriraj Hospital, Bangkok and Bamrasnaradura Hospital, Nonthaburi, Thailand, were asked to provide stool samples. At the same time, HIV-infected patients without diarrhea, who were admitted to these hospitals because of other conditions, were also asked to provide a stool sample. Overall, 166 fecal samples from 155 HIV-infected patients, collected and archived between November 1999 and May 2004, were available for this study.

### Microscopic examination of stools

Routine microscopic examination of fecal samples was performed at the Infectious Diseases and Tropical Medicine Laboratory, Department of Medicine, Faculty of Medicine Siriraj Hospital, as previously described [13]. All samples were examined by direct microscopy of a wet preparation, with or without iodine staining, and of sediments obtained by formol-ether extraction of about 2 g of feces. Sediments were analyzed by Ziehl-Neelsen staining for the detection of *Cryptosporidium* and *Isospora* oocysts, and by modified trichrome staining for the detection of *Microsporidium* spp. *Entamoeba histolytica* was not differentiated from the nonpathogenic *Entamoeba dispar*. Larvae of *Strongyloides* were detected by microscopy or by culture [13]. Culture was used to detect common enteric bacterial species, including *Salmonella*, *Shigella*, *Vibrio* and *Campylobacter*. Finally, *Clostridium difficile* toxin A was detected as reported [13].

### DNA extraction and molecular typing for *Cryptosporidium*

DNA was extracted from approximately 200 mg (or 200 μl for watery or loose samples) of stool by using the FastPrep120 apparatus and the FastDNA kit (MP Biomedicals, Santa Ana, USA). For species identification, a nested PCR assay was used to amplify a ~590 bp fragment of the small subunit rRNA (*18S* rDNA) gene, as described [14]. For subtyping at the glycoprotein 60 (*gp60*) locus, primers Gp15start (5′-ATG AGA TTG TCG CTC ATT ATC-3′) and Gp15end (5′-TTA CAA CAC GAA TAA GGC TGC-3′) were used in primary PCR to amplify a ~950 bp fragment from all samples. For the nested reaction, primers AL3531 (5′-ATA GTC TCC GCT GTA TTC-3′) and AL3534 (5′-GCA GAG GAA CCA GCA TC-3′) [15] were used for samples classified as *C. hominis* or *C. parvum*, and primers ATGFmod (5′-GAG ATT GTC GCT CGT TAT CG-3′) and GATR2 (5′-GAT TGC AAA AAC GGA AGG-3′) [16] were used for samples classified as *C. meleagridis*. For samples classified as *C. canis*, *C. felis* and *C. suis*, combinations of the nested PCR primers reported above were tested. PCR conditions, for both primary and secondary amplification, were as follows: initial denaturation at 94 °C for 3 min, followed by 40 cycles of denaturation at 94 °C for 30 s, annealing at 50 °C for 1 min and extension at 72 °C for 1 min, followed by a final extension step at 72 °C for 7 min. Negative (no template) and positive (DNA from the *C. parvum* Moredun strain) controls were included in each experiment. PCR was performed using 25 μl of 2× GoTaqGreen (Promega, Madison, USA), 10 pmol of each primer, 5.0 μl of DNA, and nuclease-free water up to a final volume of 50 μl. Reactions were performed on a Perkin Elmer 9700 apparatus (Life Technologies, Carlsbad, USA). Aliquots of PCR reactions (10 μl) were loaded on 1.5% agarose gel stained with ethidium bromide. PCR products were purified using spin columns (QiaQuick PCR purification kit, Qiagen, Milan, Italy). Bidirectional sequencing of the PCR products was performed by a commercial company (BMR Genomics, Padua, Italy). Sequences were edited and assembled using the software package SeqMan v.7.1 (DNASTAR, Madison, USA). BLASTn searches (http://blast.ncbi.nlm.nih.gov/Blast.cgi) against the GenBank database were used to identify *Cryptosporidium* at the species and subtype levels. Novel *gp60* sequences were deposited in the GenBank database under the accession numbers MK331714-MK331719.

## Results

### Epidemiological and clinical data

Among the 155 patients, 90 were male and 65 were female, with a median age of 34.5 years (range, 20–65 years) for both genders. The information collected from patients regarded the consistency of stool, duration of diarrhea, number of stool passed per day, presence of mucus and CD4^+^ T-lymphocyte count (Additional file 1: Table S1). Diarrhea lasting less than 3 weeks was observed in 52 patients, chronic diarrhea characterized 98 patients, while three patients had no diarrhea. No data about diarrhea were available for the remaining five patients. Concerning the presence of mucus, 85 had mucus and 53 patients did not have mucus in their stools; no data were available for the remaining 17 patients. Finally, the CD4^+^ T-lymphocyte count was determined in 113 patients, and the median count was 12 cell/mm^3^ (range 1–331); all patients, except two, had a CD4^+^ count of less than 200 cell/mm^3^.

### Detection of enteric pathogens

Microscopic analysis and culture methods showed that 130 of 155 (83.9%) patients had at least one detectable pathogen in the stool (Table 1). Mixed infections with two or three pathogens were detected in 34 (21.9%) and six (3.9%) patients, respectively (Table 1).

**Table 1:** Pathogens detected in the stools of 155 HIV-infected individuals

Pathogens detected	No. of patients
Positive for *Cryptosporidium*	104
Alone	70
Co-infected with *Strongyloides*	9
Co-infected with *Opisthorchis*	4
Co-infected with microsporidia	3
Co-infected with *Entamoeba*	1
Co-infected with *Giardia*	1
Co-infected with *Salmonella*	2
Co-infected with *Vibrio*	1
Co-infected with *Clostridium*	2
Co-infected with *Campylobacter*	4
Co-infected with *Aeromonas*	1
Co-infected with *Mycobacterium tuberculosis*	1
Co-infected with *Ascaris* and *Campylobacter*	1
Co-infected with *Isospora* and *Opisthorchis*	1
Co-infected with microsporidia and *Strongyloides*	1
Co-infected with microsporidia and *Campylobacter*	1
Co-infected with *Campylobacter* and *Clostridium*	1
Negative for *Cryptosporidium*	51
Microsporidia	7
*Strongyloides*	4
*Giardia*	2
*Isospora*	2
*Mycobacterium tuberculosis*	2
*Campylobacter*	2
*Clostridium*	1
Hookworm and *Salmonella*	1
Hookworm and *Campylobacter*	1
*Opisthorchis* and *Vibrio*	1
*Entamoeba* and microsporidia	1
*Isospora* and *Campylobacter*	1
Microsporidia, *Salmonella* and *Clostridium*	1
No pathogen detected	25

Protozoans detected by microscopy included *Cryptosporidium* spp. in 104 patients, *Isospora* spp. in four patients, *Giardia* spp. in three patients, and *Entamoeba histolytica/dispar* in two patients. Spores of microsporidian species were identified in 14 patients. Infection by helminths was found in 23 patients, with identification of *Strongyloides* larvae in 14 patients, of *Opisthorchis viverrini* eggs in six patients, of hookworm eggs in two patients and of *Ascaris* eggs in one patient. *Mycobacterium tuberculosis* was found in three patients. Enteric bacterial pathogens were found in 17 patients, including *Campylobacter* spp. in 10 patients, *Salmonella* spp. in two patients, *Vibrio* spp. in two patients and *Aeromonas* spp. in one patient. Finally, the *Clostridium difficile* toxin A was detected in three patients.

### Molecular identification of *Cryptosporidium* species

PCR amplification of the *18S* rDNA gene fragment identified 92 of the 155 (59%) samples as positive for *Cryptosporidium* spp. (Additional file 1: Table S1). There was a good correspondence between microscopy and PCR: 90 of the 104 (86.5%) microscopically positive samples were PCR positive, whereas only two of the 63 (4%) microscopically negative samples were PCR positive.

Sequencing of the PCR products identified *C. hominis* as the most prevalent species (*n* = 42), followed by *C. meleagridis* (*n* = 20), *C. canis* (*n* = 12), *C. felis* (*n* = 7), *C. suis* (*n* = 6) and *C. parvum* (*n* = 5) (Additional file 1: Table S1).

### Genotyping of *Cryptosporidium* at the *gp60* locus

All samples positive by *18S* rDNA PCR were tested with different combination of primers to amplify fragments of the *gp60* gene. No amplification was observed for samples classified as *C. felis*, *C. canis* and *C. suis*.

For *C. hominis*, PCR amplification was positive for 29 of the 42 isolates (69%), and sequencing revealed the presence of nine different subtypes in allelic families Ia, Ib, Id, Ie and If (Table 2). Subtype IeA11G3T3 was the most prevalent (*n* = 13), but allelic family Ia was the most diverse, with four subtypes. In particular, we identified subtypes IaA18R3, IaA19R3 and IaA20R3 in three, one and two isolates, respectively, as well as a new subtype (IaA16R3) in four isolates, which showed the highest similarity (98%) to an isolate from Ghana (KM538999). Finally, subtype IfA12G1 was detected in three isolates, whereas subtype IbA9G3, subtype IdA17 and a new subtype, IdA11, were each found in single isolates.

**Table 2:** Distribution of *gp60* subtypes in *C. hominis*, *C. meleagridis* and *C. parvum* isolates from HIV-infected patients in Thailand

Species	Allelic family	Subtype	*n*
*C. hominis*	Ia	IaA16R3	4
		IaA18R3	3
		IaA19R3	1
		IaA20R3	2
	Ib	IbA9G3	1
	Id	IdA11	1
		IdA17	1
	Ie	IeA11G3T3	13
	If	IfA12G1	3
*C. meleagridis*	IIIb	IIIbA19G1R1	1
		IIIbA20G1R1	2
		IIIbA21G1R1b	2
		IIIbA22G1R1c	1
		IIIbA23G1R1b	2
		IIIbA23G1R1c	2
		IIIbA24G1R1	1
	IIIe	IIIeA22G1R1	2
	IIIg	IIIgA19G3R1	1
*C. parvum*	IIo	IIoA16G1	4

For *C. meleagridis*, amplification yielded positive results for 14 of the 20 isolates (70%). Sequencing revealed the presence of allelic families IIIb (*n* = 13), IIIe (*n* = 2) and IIIg (*n* = 1) (Table 2). Subtypes were defined following a proposed nomenclature [16]. Accordingly, in allelic family IIIb, subtypes IIIbA19G1R1 (100% identity to KJ216111), IIIbA20G1R1 (100% identity to AB539720), IIIbA21G1R1b (100% identity to KJ210618), IIIbA22G1R1c (100% identity to KJ210614), IIIbA23G1R1b (100% identity to KJ210609), IIIbA23G1Rc (a new subtype with two nucleotide differences in the non-repeated region) and IIIbA24G1R1 (a new subtype with a single nucleotide difference in the non-repeated region), were identified. In allelic family IIIe, a new subtype, IIIeA22G1R1 (closely related to IIIeA21G2R1; KU852728), was found in two isolates. Finally, a new subtype in allelic family IIIg, IIIgA19G3R1, was found in a single isolate.

For *C. parvum*, amplification was successful for 4 out of 5 isolates, and sequencing revealed the presence of a single subtype, IIoA16G1 (100% identity to JN867335).

### *Cryptosporidium* species and subtypes in multiple samples from the same patients

Additional stool samples (*n* = 11) collected at different time points from 10 patients were studied. Of the five *C. hominis* cases, four were confirmed at the species level, and three at the subtype (IeA11g3T3) level; the remaining samples were PCR negative. The single *C. meleagridis* case was confirmed at both the species and subtype (IIIbA20G1R1) level. The two cases involving *C. canis* were confirmed at the species level, whereas the two cases involving *C. felis* were PCR negative (Additional file 2: Table S2).

## Discussion

This study initially aimed at determining the etiologic agents of diarrhea in HIV-infected people in Thailand. Microscopic analyses of stools (Table 1) showed a very high prevalence of single and mixed infections, which were sustained by a variety of enteric pathogens, in agreement with other studies [3, 5]. Infections due to *Cryptosporidium* spp. were particularly common, and this prompted a molecular study to identify the parasite species and subtypes and contribute to a better understanding of transmission routes.

The high prevalence of *C. hominis* (about 45%) is in agreement with previous studies conducted in this country [8–12] and, more generally, with findings from low income regions, where anthroponotic transmission prevails over zoonotic transmission [17–19]. Typing at the *gp60* locus showed extensive genetic variability among *C. hominis* isolates, with nine subtypes belonging to allelic families Ia, Ib, Id, Ie and If, known to be associated with human cryptosporidiosis in low income countries [18, 20–22].

The other *Cryptosporidium* species detected in this study are zoonotic, and infect different animal hosts, including livestock (*C. parvum*), avian species (*C. meleagridis*), cats (*C. felis*), dogs (*C. canis*) and pigs (*C. suis*). The circulation of these parasite species among HIV-infected people in Thailand has been previously documented [8–12]. On the other hand, the few molecular studies on animal cryptosporidiosis identified *C. canis* in two dogs, *C. felis* in two cats, *C. meleagridis* in a pigeon [23] and *C. parvum* in dairy cows [10, 24] and water buffaloes [25]. The existence of zoonotic reservoirs is therefore plausible, yet human-to-human transmission of zoonotic species cannot be excluded. Subtyping data are needed to better understand the extent of zoonotic transmission.

The majority of the *C. meleagridis* subtypes identified in this study had been previously reported in patients who had travelled, immediately prior to the infection, to Thailand, Vietnam and/or India [16], or had acquired infection in Japan and China [26, 27]. Zoonotic transmission of *C. meleagridis* to humans has been demonstrated in several studies [16, 28], and specific *gp60* subtypes were found to be shared by humans and birds [29], including the IIIbA22G1R1b and IIIbA22G1R1c subtypes identified in the present study.

Similar to *C. meleagridis*, the subtype IIoA16G1 identified in four *C. parvum* samples was previously described in a single patient from Sweden, who was reported to have traveled to Thailand before developing the symptoms [30]. This suggests that specific subtypes of different species play a more important role than others in defined regions of the world, underlying the complex epidemiology of cryptosporidiosis [18].

Mixed infections were found in about a quarter of the patients, and in one third of patients infected with *Cryptosporidium*. This is in line with findings from other studies [31], and should be taken into account when attributing severity and duration of diarrheal disease to specific pathogens.

This study has several limitations. First, the samples were collected at a time when antiretroviral therapies were not widely available for HIV-infected patients; it is possible that the relative prevalence of diarrhea-causing pathogens, including *Cryptosporidium*, has changed after the implementation of free-of-charge therapy for HIV-infected patients in Thailand. The retrospective nature did not allow the collection of further samples, or of clinical and epidemiologic data. It is therefore difficult to contextualize the results of this retrospective study and assessed rigorously for their current relevance. However, the temporal dynamics of cryptosporidiosis has not been carefully investigated, and genotyping data, even if from archived samples, are important. Secondly, a not negligible proportion (13.5%) of the samples was PCR negative, possibly due to a negative impact of the prolonged storage of stools on DNA integrity. Thirdly, and despite the use of combinations of published primers, no amplification of the *gp60* gene could be obtained for *C. felis*, *C. canis* and *C. suis* isolates, which accounted for about one quarter of infections. Subtyping methods for these species should be developed.

## Conclusions

This retrospective study highlights the complex epidemiology of *Cryptosporidium* infection in the highly vulnerable population of HIV-infected individuals. Extensive genetic heterogeneity was found among *C. hominis* and *C. meleagridis* isolates, with many subtypes previously identified in travelers returning from Thailand or other Asiatic countries.

## Additional files

**Additional file 1: Table S1.** List of the samples included in the study, with clinical, epidemiological and laboratory data.

**Additional file 2: Table S2.** Detection of* Cryptosporidium* species and subtypes in additional samples collected at different time points from HIV-infected patients.

## Abbreviations

HIV: Human Immunodeficiency Virus
AIDS: Acquired Immune Deficiency Syndrome
