# Supplementary material for: A retrospective molecular study of Cryptosporidium species and genotypes in HIV-infected patients from Thailand
Source: Parasit Vectors. 2019 Mar 12;12:91. doi: 10.1186/s13071-019-3348-4 (PMC6417249; doi:10.1186/s13071-019-3348-4)
Supplement: Supplementary file 1 — Additional file 1: Table S1. List of the samples included in the study, with clinical, epidemiological and laboratory data. [file 13071_2019_3348_MOESM1_ESM.docx]

**Additional file 1: Table S1.** List of the samples included in the study, with clinical, epidemiological and laboratory data.

| **Sample code** | **Sex** | **Age** | **Stool consistency** | **Duration of diarrhoea (days)** | **Presence of mucus** | **N. of stool per day** | **CD4 count (mm^3^)** | **Microscopy for** *Cryptosporidium* | **Other pathogens identified** | ***Cryptosporidium* species** | ***Cryptosporidium* gp60 subtype** |
| --- | --- | --- | --- | --- | --- | --- | --- | --- | --- | --- | --- |
| BAMDI 191 | F | 35 | Watery | 1 | Yes | 3 | 8 | Positive |  | *C. canis* | Negative |
| BAMDI 314 | M | 51 | Semiformed | 4 | No | 6 | 4 | Positive |  | *C. canis* | Negative |
| BAMDI 334 | F | 34 | Watery | 14 | Yes | 5 | 14 | Positive | Strongyloides | *C. canis* | Negative |
| BAMDI 339 | F | 25 | Watery | 90 | Yes | 4 | 9 | Positive |  | *C. canis* | Negative |
| BAMDI 345 | M | 40 | Watery | 7 | Yes | 10 | 14 | Positive | Opisthorchis | *C. canis* | Negative |
| BAMDI 349 | F | 65 | Watery | 3 | No | 6 | n.d. | Positive | Strongyloides | *C. canis* | Negative |
| BAMDI 351 | F | 29 | Watery | 30 | No | 5 | 7 | Positive | Strongyloides | *C. canis* | Negative |
| HIVDI 084 | F | 35 | Watery | 30 | Yes | 5 | 84 | Positive |  | *C. canis* | Negative |
| HIVDI 236 | M | 31 | Watery | 90 | Yes | 10 | 8 | Positive |  | *C. canis* | Negative |
| HIVDI 557 | M | 28 | Watery | 180 | Yes | 6 | 35 | Positive |  | *C. canis* | Negative |
| HIVDI 590 | M | 27 | Watery | 60 | No | 4 | 26 | Positive |  | *C. canis* | Negative |
| HIVDI 989 | M | 34 | Watery | 120 | Yes | 5 | 4 | Positive |  | *C. canis* | Negative |
| BAMDI 293 | M | 45 | Watery | 4 | No | 4 | 9 | Positive |  | *C. felis* | Negative |
| BAMDI 329 | F | 30 | Watery | 30 | Yes | 3 | 7 | Positive | Strongyloides | *C. felis* | Negative |
| BAMDI 330 | M | 33 | Watery | 180 | Yes | 4 | 50 | Positive |  | *C. felis* | Negative |
| BAMDI 381 | M | 26 | Watery | 60 | Yes | 6 | n.d. | Positive | Strongyloides | *C. felis* | Negative |
| HIVDI 077 | F | 27 | Watery | 90 | Yes | 8 | n.d. | Positive | Strongyloides | *C. felis* | Negative |
| HIVDI 400 | M | 32 | Watery | 30 | No | 4 | 23 | Positive |  | *C. felis* | Negative |
| HIVDI 820 | F | 40 | Watery | n.d. | Yes | n.d. | 31 | Positive | Salmonella | *C. felis* | Negative |
| 1644000161 | M | 26 | Watery | 14 | No | 5 | 4 | Positive |  | *C. hominis* | Negative |
| BAMDI 006 | F | 34 | Watery | 60 | No | 1 | n.d. | Positive |  | *C. hominis* | IaA16R3 |
| BAMDI 008 | F | 32 | Watery | 30 | Yes | 3 | 6 | Positive |  | *C. hominis* | IeA11G3T3 |
| BAMDI 024 | M | 39 | Watery | 10 | Yes | 1 | n.d. | Positive | Vibrio | *C. hominis* | Negative |
| BAMDI 025 | F | 49 | Watery | 5 | No | 3 | n.d. | Positive |  | *C. hominis* | Negative |
| BAMDI 065 | F | 21 | Watery | 21 | No | 9 | 14 | Positive |  | *C. hominis* | IaA16R3 |
| BAMDI 088 | F | 26 | Watery | 60 | No | 10 | 9 | Positive | Campylobacter | *C. hominis* | IaA16R3 |
| BAMDI 096 | F | 32 | Watery | 60 | No | 6 | 16 | Positive |  | *C. hominis* | IbA9G3 |
| BAMDI 115 | M | 42 | Watery | 30 | Yes | 10 | 7 | Positive |  | *C. hominis* | IeA11G3T3 |
| BAMDI 143 | F | 27 | Watery | 120 | Yes | 5 | 2 | Positive |  | *C. hominis* | IeA11G3T3 |
| BAMDI 157 | F | 23 | Watery | 4 | Yes | 8 | 7 | Positive |  | *C. hominis* | IeA11G3T3 |
| BAMDI 239 | F | 29 | Watery | 60 | Yes | 10 | 24 | Positive |  | *C. hominis* | Negative |
| BAMDI 261 | M | 44 | Watery | 10 | Yes | 4 | n.d. | Positive | Microsporidium | *C. hominis* | IfA12G1 |
| BAMDI 266 | M | 28 | Watery | 5 | Yes | 3 | n.d. | Positive |  | *C. hominis* | Negative |
| BAMDI 285 | F | 37 | Watery | 90 | Yes | 6 | 9 | Positive | Aeromonas | *C. hominis* | IeA11G3R3 |
| BAMDI 300 | F | 28 | Watery | 21 | Yes | 5 | 2 | Positive |  | *C. hominis* | IaA18R3 |
| BAMDI 311 | M | 38 | Watery | 60 | Yes | 5 | n.d. | Positive | Ascaris, Campylobacter | *C. hominis* | IeA11G3R3 |
| BAMDI 325 | F | 35 | Watery | 90 | Yes | 10 | 40 | Positive |  | *C. hominis* | Negative |
| BAMDI 331 | F | 45 | Watery | 90 | Yes | 5 | 52 | Positive |  | *C. hominis* | IeA11G3R3 |
| BAMDI 360 | M | 32 | Watery | 30 | Yes | 3 | 1 | Positive |  | *C. hominis* | IdA17 |
| BAMDI 379 | M | 35 | Watery | 14 | Yes | 4 | n.d. | Positive |  | *C. hominis* | IeA11G3T3 |
| BAMDI 411 | M | 30 | Watery | 60 | Yes | 8 | n.d. | Positive |  | *C. hominis* | IeA11G3T3 |
| BAMDI 449 | F | 27 | Watery | 150 | Yes | 3 | 20 | Positive |  | *C. hominis* | IaA18R3 |
| BAMDI 454 | F | 40 | Watery | 30 | Yes | 4 | 32 | Positive |  | *C. hominis* | IfA12G1 |
| HIVDI 007 | M | 34 | Watery | 150 | Yes | 8 | 10 | Positive |  | *C. hominis* | Negative |
| HIVDI 067 | F | 45 | Watery | n.d. | Yes | n.d. | n.d. | Positive |  | *C. hominis* | IaA19R3 |
| HIVDI 070 | F | 52 | Watery | 210 | Yes | 5 | n.d. | Positive | Clostridium | *C. hominis* | IaA20R3 |
| HIVDI 073 | F | 21 | Watery | 30 | Yes | 4 | 28 | Positive | Strongyloides | *C. hominis* | IaA20R3 |
| HIVDI 090 | M | n.d. | Watery | n.d. | n.d. | n.d. | n.d. | Positive |  | *C. hominis* | IeA11G3T3 |
| HIVDI 136 | F | 35 | Watery | 4 | No | 4 | 8 | Positive |  | *C. hominis* | IeA11G3T3 |
| HIVDI 156 | M | 23 | Watery | 30 | Yes | 7 | 1 | Positive |  | *C. hominis* | Negative |
| HIVDI 274 | M | 28 | Watery | 60 | No | 3 | n.d. | Positive | Strongyloides | *C. hominis* | Negative |
| HIVDI 500 | M | 33 | Watery | 120 | Yes | 4 | 13 | Positive | Giardia | *C. hominis* | IdA11 |
| HIVDI 529 | M | 30 | Semiformed | no diarrhea | Yes | 0 | 9 | Positive |  | *C. hominis* | Negative |
| HIVDI 613 | F | n.d. | Watery | 30 | Yes | 2 | 55 | Positive |  | *C. hominis* | IaA18R3 |
| HIVDI 630 | M | 35 | Watery | 90 | No | 5 | 3 | Positive |  | *C. hominis* | IeA11G3T3 |
| HIVDI 704 | F | 40 | Watery | 60 | Yes | 4 | 33 | Positive |  | *C. hominis* | Negative |
| HIVDI 783 | M | 35 | Watery | 30 | n.d. | 3 | n.d. | Negative | Negative | *C. hominis* | Negative |
| HIVDI 819 | M | 44 | Watery | 180 | Yes | 5 | 9 | Positive |  | *C. hominis* | IfA12G1 |
| HIVDI 1051 | M | 26 | Watery | 7 | No | 8 | 39 | Positive | Strongyloides, Microsporidium | *C. hominis* | IaA16R3 |
| HIVDI 1060 | F | n.d. | Watery | 30 | No | 10 | 11 | Positive |  | *C. hominis* | IeA11G3T3 |
| HIVDI 1110 | F | 34 | Watery | 120 | No | 5 | 14 | Positive | Salmonella | *C. hominis* | Negative |
| BAMDI 036 | F | 33 | Watery | 30 | No | 8 | 12 | Positive | Campylobacter, Clostridium | *C. meleagridis* | IIIbA20G1R1 |
| BAMDI 076 | F | 41 | Watery | 90 | Yes | 3 | 2 | Positive |  | *C. meleagridis* | Negative |
| BAMDI 267 | M | 47 | Watery | 2 | Yes | 10 | 35 | Positive | Opisthorchis | *C. meleagridis* | IIIbA21G1R1c |
| BAMDI 373 | M | 37 | Watery | 150 | Yes | 5 | 3 | Positive |  | *C. meleagridis* | Negative |
| BAMDI 402 | F | 27 | Watery | 30 | Yes | 5 | n.d. | Positive |  | *C. meleagridis* | IIIbA21G1R1b |
| HIVDI 034 | M | 22 | Watery | 150 | No | 6 | 6 | Positive |  | *C. meleagridis* | IIIbA23G1R1b |
| HIVDI 039 | M | 28 | Watery | 45 | No | 3 | 1 | Positive |  | *C. meleagridis* | IIIgA19G3R1 |
| HIVDI 047 | M | 34 | Watery | 90 | No | 10 | n.d. | Negative |  | *C. meleagridis* | IIIbA23G1R1c |
| HIVDI 202 | F | 26 | Watery | 14 | Yes | 4 | 4 | Positive |  | *C. meleagridis* | Negative |
| HIVDI 230 | M | 34 | Watery | 365 | No | 3 | 4 | Positive | Entamoeba histolytica/dispar | *C. meleagridis* | IIIbA19G1R1 |
| HIVDI 508 | F | 28 | Watery | n.d. | No | n.d. | 59 | Positive |  | *C. meleagridis* | Negative |
| HIVDI 553 | M | 36 | Watery | 30 | Yes | 4 | 3 | Positive |  | *C. meleagridis* | IIIbA22G1R1c |
| HIVDI 585 | M | 28 | Watery | 120 | No | 5 | n.d. | Positive |  | *C. meleagridis* | IIIbA23G1R1c |
| HIVDI 598 | M | 30 | Watery | 7 | Yes | 2 | 3 | Positive | Opisthorchis | *C. meleagridis* | IIIbA20G1R1c |
| HIVDI 600 | M | 37 | Watery | 1 | No | 3 | 9 | Positive | Opisthorchis | *C. meleagridis* | Negative |
| HIVDI 659 | F | n.d. | Watery | 60 | Yes | 10 | 9 | Positive | Campylobacter | *C. meleagridis* | IIIbA24G1R1 |
| HIVDI 697 | F | 38 | Watery | 120 | Yes | 6 | 23 | Positive |  | *C. meleagridis* | IIIbA23G1R1 |
| HIVDI 721 | F | 34 | Watery | 60 | No | 2 | n.d. | Positive | Campylobacter | *C. meleagridis* | IIIeA22G2R1 |
| HIVDI 888 | F | 29 | Watery | 14 | Yes | 4 | 10 | Positive |  | *C. meleagridis* | IIIeA22G1R1 |
| HIVDI 962 | F | 34 | Watery | 30 | Yes | 4 | 139 | Positive | Strongyloides | *C. meleagridis* | Negative |
| BAMDI 121 | F | 24 | Watery | 7 | Yes | 4 | 10 | Positive |  | *C. parvum* | IIoA16G1 |
| HIVDI 155 | M | 34 | Watery | 60 | Yes | 10 | 10 | Positive |  | *C. parvum* | IIoA16G1 |
| HIVDI 711 | F | 39 | Watery | 210 | No | 4 | n.d. | Positive |  | *C. parvum* | Negative |
| HIVDI 712 | M | 62 | Watery | 21 | Yes | 5 | 14 | Positive |  | *C. parvum* | IIoA16G1 |
| HIVDI 854 | M | 43 | Watery | 14 | Yes | 3 | 7 | Positive | Isospora, Opisthorchis | *C. parvum* | IIoA16G1 |
| BAMDI 007 | M | 39 | Watery | 210 | Yes | 10 | 1 | Positive |  | *C. suis* | Negative |
| HIVDI 120 | M | 39 | Watery | 90 | No | 6 | 8 | Positive |  | *C. suis* | Negative |
| HIVDI 147 | F | 27 | Watery | 3 | No | 2 | 7 | Positive | Clostridium | *C. suis* | Negative |
| HIVDI 240 | M | 27 | Watery | 21 | Yes | 5 | 55 | Positive |  | *C. suis* | Negative |
| HIVDI 421 | F | 28 | Watery | 30 | No | 5 | 4 | Positive | Microsporidium, Campylobacter | *C. suis* | Negative |
| HIVDI 961 | M | 35 | Watery | 60 | No | 4 | 9 | Positive |  | *C. suis* | Negative |
| BAMDI 002 | M | 29 | Watery | 180 | No | 4 | n.d. | Negative | Strongyloides | Negative | n.d. |
| BAMDI 020 | M | 42 | Watery | 5 | No | 2 | n.d. | Negative |  | Negative | n.d. |
| BAMDI 030 | F | 38 | Watery | 90 | Yes | 8 | n.d. | Negative | Isospora, Campylobacter | Negative | n.d. |
| BAMDI 031 | F | 47 | Watery | 2 | No | 10 | n.d. | Negative |  | Negative | n.d. |
| BAMDI 037 | M | 42 | Watery | 30 | Yes | 10 | 40 | Negative |  | Negative | n.d. |
| BAMDI 044 | M | 37 | Watery | 30 | Yes | 8 | n.d. | Negative |  | Negative | n.d. |
| BAMDI 084 | M | 28 | Watery | 60 | n.d. | 7 | 15 | Positive | Microsporidium | Negative | n.d. |
| BAMDI 110 | M | 43 | Watery | 7 | Yes | 3 | 68 | Negative |  | Negative | n.d. |
| BAMDI 198 | M | 35 | Watery | 90 | Yes | 6 | 30 | Negative | Microsporidium | Negative | n.d. |
| BAMDI 207 | M | 39 | Watery | 90 | No | 3 | n.d. | Negative |  | Negative | n.d. |
| BAMDI 223 | F | 29 | Watery | 30 | No | 3 | 4 | Negative | Microsporidium | Negative | n.d. |
| BAMDI 263 | F | 46 | Watery | 4 | n.d. | 3 | 16 | Positive |  | Negative | n.d. |
| BAMDI 268 | M | 34 | Semiformed | 180 | n.d. | 3 | 9 | Positive |  | Negative | n.d. |
| BAMDI 332 | M | 32 | Watery | 3 | n.d. | 3 | n.d. | Negative | Opisthorchis, Vibrio | Negative | n.d. |
| BAMDI 335 | M | 48 | Watery | 14 | Yes | 5 | 17 | Negative |  | Negative | n.d. |
| BAMDI 355 | M | 41 | Watery | 180 | No | 2 | 10 | Negative |  | Negative | n.d. |
| BAMDI 358 | M | 36 | Watery | 2 | n.d. | 5 | 15 | Positive |  | Negative | n.d. |
| BAMDI 370 | F | 42 | Watery | 7 | Yes | 7 | 94 | Negative | Microsporidium | Negative | n.d. |
| BAMDI 371 | M | 41 | Watery | 120 | Yes | 10 | 5 | Negative | Mycobacterium | Negative | n.d. |
| BAMDI 401 | M | 30 | Watery | 30 | n.d. | 3 | n.d. | Positive |  | Negative | n.d. |
| BAMDI 408 | M | 41 | Semiformed | 120 | Yes | 6 | n.d. | Negative |  | Negative | n.d. |
| BAMDI 415 | M | 42 | Watery | 10 | Yes | 5 | 63 | Negative |  | Negative | n.d. |
| BAMDI 429 | F | 38 | Watery | 30 | n.d. | 4 | 11 | Positive | Campylobacter | Negative | n.d. |
| HIVDI 028 | F | 33 | Watery | 210 | No | 6 | 7 | Negative | Microsporidium, Salmonella, Clostridium | Negative | n.d. |
| HIVDI 041 | M | 42 | Watery | 7 | No | 5 | 25 | Positive |  | Negative | n.d. |
| HIVDI 051 | F | 20 | Watery | 365 | No | 3 | 8 | Negative | Microsporidium | Negative | n.d. |
| HIVDI 052 | F | 31 | Watery | 15 | Yes | 6 | n.d. | Negative | Hookworm, Salmonella | Negative | n.d. |
| HIVDI 065 | M | 25 | Watery | 180 | Yes | 5 | n.d. | Negative |  | Negative | n.d. |
| HIVDI 076 | F | 46 | Watery | 30 | No | 10 | 83 | Positive |  | Negative | n.d. |
| HIVDI 087 | M | 32 | Watery | 30 | No | 2 | 14 | Negative | Campylobacter | Negative | n.d. |
| HIVDI 091 | M | 28 | Watery | 365 | n.d. | 3 | 15 | Negative | Microsporidium | Negative | n.d. |
| HIVDI 095 | F | 23 | Semiformed | 30 | No | 3 | 69 | Negative | Campylobacter | Negative | n.d. |
| HIVDI 097 | M | 27 | Watery | 210 | Yes | 3 | 110 | Negative |  | Negative | n.d. |
| HIVDI 104 | M | 28 | Watery | 10 | Yes | 6 | 11 | Negative | Clostridium | Negative | n.d. |
| HIVDI 108 | M | 36 | Watery | 3 | No | 3 | n.d. | Negative | Mycobacterium | Negative | n.d. |
| HIVDI 122 | M | 30 | Watery | n.d. | No | 4 | 46 | Negative | Isospora | Negative | n.d. |
| HIVDI 210 | M | 29 | Watery | 60 | Yes | 4 | 15 | Negative | Strongyloides | Negative | n.d. |
| HIVDI 215 | M | 37 | Soft | no diarrhea | No | n.d. | 19 | Negative |  | Negative | n.d. |
| HIVDI 233 | M | 36 | Watery | 180 | Yes | 3 | n.d. | Negative | Isospora | Negative | n.d. |
| HIVDI 244 | F | 45 | Watery | 60 | No | 2 | 284 | Negative |  | Negative | n.d. |
| HIVDI 252 | F | 40 | Watery | 21 | No | 6 | n.d. | Positive |  | Negative | n.d. |
| HIVDI 437 | M | 34 | Soft | no diarrhea | Yes | 0 | 37 | Negative |  | Negative | n.d. |
| HIVDI 464 | M | 38 | Watery | 30 | Yes | 4 | 12 | Negative | Entamoeba histolytica/dispar, Microsporidium | Negative | n.d. |
| HIVDI 517 | M | 33 | Watery | 14 | No | 3 | n.d. | Negative |  | Negative | n.d. |
| HIVDI 531 | F | 36 | Watery | 90 | Yes | 10 | n.d. | Negative | Strongyloides | Negative | n.d. |
| HIVDI 532 | M | 39 | Watery | 1 | Yes | 2 | n.d. | Negative |  | Negative | n.d. |
| HIVDI 540 | M | 32 | Watery | 60 | Yes | 3 | 331 | Negative | Hookworm, Campylobacter | Negative | n.d. |
| HIVDI 550 | F | 26 | Watery | 14 | Yes | 5 | n.d. | Negative | Giardia | Negative | n.d. |
| HIVDI 555 | F | 25 | Watery | 14 | n.d. | 4 | n.d. | Positive | Mycobacterium | Negative | n.d. |
| HIVDI 577 | M | 41 | Watery | 60 | No | 3 | 74 | Negative | Microsporidium | Negative | n.d. |
| HIVDI 654 | M | 34 | Watery | 365 | n.d. | 3 | 39 | Positive |  | Negative | n.d. |
| HIVDI 684 | M | 28 | Watery | 1 | n.d. | 1 | 53 | Positive | Microsporidium | Negative | n.d. |
| HIVDI 699 | M | 43 | Watery | 30 | n.d. | 6 | 9 | Positive |  | Negative | n.d. |
| HIVDI 754 | M | 24 | Watery | 30 | No | 4 | n.d. | Negative |  | Negative | n.d. |
| HIVDI 784 | M | 31 | Watery | 365 | No | 3 | 12 | Negative |  | Negative | n.d. |
| HIVDI 801 | M | 36 | Watery | 90 | Yes | 3 | 10 | Negative | Microsporidium | Negative | n.d. |
| HIVDI 846 | F | 44 | Watery | 7 | Yes | 2 | 6 | Negative | Giardia | Negative | n.d. |
| HIVDI 850 | M | 35 | Watery | 15 | Yes | n.d. | 11 | Negative |  | Negative | n.d. |
| HIVDI 909 | F | 57 | Watery | 4 | Yes | 4 | 188 | Negative |  | Negative | n.d. |
| HIVDI 944 | M | 36 | Watery | 90 | Yes | 3 | 102 | Negative | Strongyloides | Negative | n.d. |
| HIVDI 957 | M | 26 | Watery | 30 | n.d. | 1 | 24 | Positive |  | Negative | n.d. |
| HIVDI 998 | M | 24 | Watery | 30 | n.d. | 1 | 19 | Negative |  | Negative | n.d. |
| HIVDI 1037 | F | 27 | Watery | 2 | No | 8 | n.d. | Negative |  | Negative | n.d. |

n.d., not determined
