# Supplementary material for: A retrospective molecular study of Cryptosporidium species and genotypes in HIV-infected patients from Thailand
Source: Parasit Vectors. 2019 Mar 12;12:91. doi: 10.1186/s13071-019-3348-4 (PMC6417249; doi:10.1186/s13071-019-3348-4)
Supplement: Supplementary file 2 — Additional file 2: Table S2. Detection of Cryptosporidium species and subtypes in additional samples collected at different time points from HIV-infected patients. [file 13071_2019_3348_MOESM2_ESM.docx]

**Additional file 2: Table S2.** Detection of *Cryptosporidium* species and subtypes in additional samples collected at different time points from HIV-infected patients.

| Sample code | Collection date | *Cryptosporidium* species | *Cryptosporidium* gp60 subtype |
| --- | --- | --- | --- |
| BAMDI 325 | 02/19/2002 | *C. hominis* | Negative |
|  | 02/20/2002 | Negative | Negative |
|  |  |  |  |
| BAMDI 008 | 02/23/2000 | *C. hominis* | IeA11G3R3 |
|  | 04/18/2000 | *C. hominis* | IeA11G3R3 |
|  |  |  |  |
| BAMDI 331 | 02/27/2002 | *C. hominis* | IeA11G3R3 |
|  | 03/15/2002 | *C. hominis* | IeA11G3R3 |
|  |  |  |  |
| BAMDI 449 | 05/19/2003 | *C. hominis* | IaA18R3 |
|  | 07/18/2003 | *C. hominis* | Negative |
|  |  |  |  |
| HIVDI 136 | 03/14/2000 | *C. hominis* | IeA11G3R3 |
|  | 11/29/2000 | *C. hominis* | IeA11G3R3 |
|  | 03/14/2001 | *C. hominis* | IeA11G3R3 |
|  |  |  |  |
| HIVDI 598 | 09/03/2001 | *C. meleagridis* | IIIbA20G1R1 |
|  | 01/24/2002 | *C. meleagridis* | IIIbA20G1R1 |
|  |  |  |  |
| BAMDI 314 | 01/07/2002 | *C. canis* | Negative |
|  | 02/12/2002 | *C. canis* | Negative |
|  |  |  |  |
| BAMDI 339 | 03/25/2002 | *C. canis* | Negative |
|  | 07/30/2002 | *C. canis* | Negative |
|  |  |  |  |
| BAMDI 329 | 02/19/2002 | *C. felis* | Negative |
|  | 10/29/2002 | Negative | Negative |
|  |  |  |  |
| BAMDI 293 | 10/09/2001 | *C. felis* | Negative |
|  | 11/02/2001 | Negative | Negative |
